# Efficacy and Safety of Intramuscular Insulin Lispro vs. Continuous Intravenous Regular Insulin for the Treatment of Dogs With Diabetic Ketoacidosis

**DOI:** 10.3389/fvets.2020.559008

**Published:** 2020-10-16

**Authors:** Eleonora Malerba, Federica Alessandrini, Giorgio Grossi, Massimo Giunti, Federico Fracassi

**Affiliations:** Department of Veterinary Medical Sciences, University of Bologna, Bologna, Italy

**Keywords:** rapid-acting insulin analogs, canine, DKA (diabetic ketoacidosis), insulin protocol, therapy, management

## Abstract

The use of rapid-acting insulin analogs as routes of administration other than IV has never been described for the treatment of dogs with diabetic ketoacidosis (DKA). This study aims to compare the efficacy and safety of a new protocol based on IM administration of insulin lispro with that of low-dose IV continuous rate infusion of regular insulin in the treatment of canine DKA. Client-owned dogs with naturally occurring DKA were included. Dogs treated with IM insulin lispro (Group L, *n* = 11) received 0.25 U/kg. The goal was to achieve a drop of at least 10% in blood glucose between 1 h and the next. If this goal was not achieved, the insulin dose was repeated hourly; otherwise, the insulin dose was not repeated up to a maximum of 3 h, after which the insulin dose was repeated anyway. When blood glucose was ≤250 mg/dL, the insulin dose was reduced to 0.125 U/kg IM every 3 h. Cases receiving IV continuous rate infusion of regular insulin (Group R, *n* = 13) were treated according to a previously published protocol. The median time to resolution of ketosis was significantly shorter in Group L (12 h; range, 4–27 h) compared to Group R (23 h; 10–46 h; *P* = 0.04). The median times to resolution of acidemia and ketoacidosis were 13 h (4–35 h) and 17.5 h (4–35 h) in Group L, and 22 h (9–80 h) and 23.5 h (10–80 h) in Group R, respectively. These differences were not significant (*P* = 0.06 and *P* = 0.09, respectively). The median length of hospitalization did not differ significantly between groups (*P* = 0.67). There were no differences in the frequency and severity of adverse events (hypoglycemia, hypokaliemia, and hypophosphatemia) between groups. The new protocol based on IM administration of insulin lispro preliminarily appears effective and safe for treatment of canine DKA.

## Introduction

Insulin therapy is the cornerstone of the treatment of diabetic ketoacidosis (DKA). In the veterinary literature, several protocols based on the use of regular crystalline insulin administered intravenously, intramuscularly or subcutaneously have been described (1–4). Although all routes of administration have been shown to be effective (5, 6), in humans continuous low-dosage IV infusion of regular insulin has been the procedure of choice because of the potential delay in the onset of action and the longer half-life of SC administration which increases the risk and occurrence of hypoglycemic events (7–9). The erratic and inconstant absorption of IM and SC insulin depends not only on the patient's hydration status (5), but also on the structural properties of the regular insulin molecule. In fact, regular insulin is a solution of zinc-insulin hexamers, whose absorption requires dissociation after injection. The process of dissociation delays the onset of action and prolongs the duration of the hypoglycemic effect (10, 11), resulting in a more variable time-action profile and in a less predictable glycemic trend, with greater risk of hypoglycemic events. Rapid-acting analogs (insulin lispro, insulin aspart, and insulin glulisine) are genetically engineered molecules designed to overcome the limitations of regular insulin (10–15). Their structure, which differs from the original molecule by one or two amino acids, hinders the hexamerization speeding up their absorption and elimination following SC injection, ensuring a rapid onset and a short duration of hypoglycemic activity (11, 16, 17). Pharmacodynamic studies in humans have shown that, following SC injection, rapid-acting analogs have an onset of action ranging from 5 to 20 min, and a duration of effect of 2 to 6 h, with peak action occurring between 30 and 90 min (10, 11). These qualities have led to the hypothesis that rapid-acting analogs could be successfully used for the management of patients with DKA. Several studies have shown that the intermittent SC injection of insulin aspart and insulin lispro was as effective as IV regular insulin in the treatment of humans with mild-to-moderate DKA (18–21). The success of insulin analogs has gradually reduced the use of regular insulin (https://investor.lilly.com/financial-information/annual-reports).

Four studies have demonstrated that IV continuous rate infusion (IVCRI) of insulin lispro or aspart is safe and appears to be as effective as an IVCRI of regular insulin for the treatment of canine and feline DKA (22–25). However, the limitation of these studies was the use of the IV route of administration, not taking advantage of the qualities deriving from the molecular structure which characterizes insulin analogs.

The aim of this study was to compare the efficacy and safety of a new protocol based on the IM administration of insulin lispro with that of low-dose IVCRI of regular insulin in the treatment of dogs with DKA. The hypothesis was that IM administration of insulin lispro is safe in treating canine DKA and that resolution of hyperglycemia, ketosis, and acidemia would be equally fast or faster in dogs treated with IM insulin lispro compared to dogs treated with IVCRI of regular insulin.

## Materials and Methods

### Dogs

Client-owned dogs with naturally occurring DKA, either newly diagnosed with diabetes mellitus (DM) or with known DM, which were admitted to the Veterinary Teaching Hospital of the University of Bologna between April 2015 and August 2019 were included in the study. Dogs which had experienced more than one episode of DKA during the study period were included in the analyses considering each hospitalization as a separate case. The diagnosis of DKA was based on the presence of clinical signs suggestive of DKA (e.g., polyuria/polydipsia, weight loss, anorexia, severe lethargy, vomiting, dehydration), blood glucose (BG) concentration >250 mg/dL, blood β-hydroxybutyrate (BHB) concentration >2.5 mmol/L, and venous pH <7.3 and/or bicarbonate <15 mEq/L. In dogs that at admission had BG concentrations above the detection limit of the portable blood glucose meters used (500 mg/dL for Optium Xceed, Abbott, UK; 750 mg/dL for AlphaTRAK, Zoetis, USA), baseline glycemia was obtained by serum biochemistry profile. Dogs with known DM with both compatible clinical signs and ketoacidosis status that received insulin therapy despite being sick, were included although not hyperglycemic. Dogs with DKA admitted between April 2015 and May 2017, treated according to a modified previously published protocol using IVCRI of regular insulin (Humulin R, Eli Lilly and Co, Sesto Fiorentino, IT) (3), were retrospectively included as a control group (Group R). From June 2018 to August 2019 dogs with DKA were treated with a new protocol based on the IM administration of insulin lispro (Humalog, Eli Lilly and Co, Sesto Fiorentino, IT) (Group L).

Dogs were excluded if they died or were euthanized before the insulin protocol was started, if the venous pH was <7.0 because this pH is associated with mortality in dogs (26), or if they had a hyperglycemic hyperosmolar syndrome. This latter condition was excluded if osmolality was <350 mOsm/kg and if a significant ketosis was present.

At the time of admission, history, physical examination findings, results of a venous blood gas analysis, complete blood count, serum biochemistry profile, urinalysis and bacterial culture from urine collected *via* cystocentesis were carried out on each dog. Other diagnostic tests, including abdominal ultrasound and thoracic radiographs, were also carried out according to the clinician's discretion in order to identify any concurrent disorder (e.g., acute pancreatitis, neoplasia).

The Scientific Ethics Committee of the University of Bologna approved this study, and the owners signed a written informed consent form before enrollment.

### Treatment Protocol

At the time of admission, the degree of dehydration was assessed subjectively (based on mucous membrane moisture, skin turgor, sunken eyes) by the emergency clinician. The initial IV fluid rate and bolus rate with crystalloids (Ringer's lactate or acetate or 0.9% NaCl) were determined based on the dog's body weight, the estimated percentage of dehydration, estimated ongoing fluid losses and maintenance fluid requirements. The formula used to calculate the fluid deficit, based on estimation of dehydration (with % dehydration expressed as decimal, e.g., 10% = 0.1), is:

$$\begin{array}{l} \text{Fluid deficit }=\%\text{dehydration }\times \text{ body weight }\left(\text{kg}\right)\;\times \;1,000\text{mL}/\text{kg} \end{array}$$

We have chosen to gradually replace this deficit over 24 h while also supplying maintenance fluid needs and matching ongoing losses. Once out of this critical phase, a fluid rate of 1.5–2 times maintenance (i.e., 60–100 mL/kg/24 h) was chosen initially with subsequent adjustments based on patient's condition. If, at the time of admission, the dog was in shock, boluses of fluids were administered at 5–10 mL/kg in 20 min until the heart rate and blood pressure were adequate.

Insulin protocols commenced after a minimum of 1 h to a maximum of 9 h after initiation of fluid therapy, once the patient's hydration status had improved and when the BG concentration was below the high detection limit of the portable blood glucose meters used, thus allowing correct monitoring of the glycemic trend. On average, insulin therapy was delayed by 2–4 h, but if within 9 h the dog was still severely or moderately dehydrated and/or BG exceeded the detection limit of glucometer, the insulin protocol was still started.

The dogs in Group L with a BG concentration >250 mg/dL received IM insulin lispro at an initial dose of 0.25 U/kg (1); the glycemia was monitored hourly. The goal was to achieve a drop of at least 10% in BG concentration between 1 h and the next. If this goal was not achieved, the insulin dose was repeated hourly, alternating the right and the left thighs; otherwise, if this goal was achieved, the insulin dose was not repeated up to a maximum of 3 h, after which the insulin dose was repeated anyway. When the BG concentration reached 250 mg/dL, the insulin dose was reduced to 0.125 U/kg IM every 3 h, and the IV fluids were changed to dextrose, starting from a 2.5% solution and using a 5 or 7.5% solution, depending on patient's response, in order to keep the BG concentrations between 150 and 300 mg/dL, until resolution of the DKA. In dogs that, at the time at which insulin protocol were initiated (“time zero”), had BG concentration ≤250 mg/dL, the protocol provided for the administration of 0.125 U/kg IM every 3 h, and the supplementation of IV fluid therapy with dextrose, as indicated above. If hypoglycemia had occurred (BG concentration <80 mg/dL) or the dog had been symptomatic for hypoglycemia, 0.25–0.5 g/kg of 50% dextrose would have been administered IV and insulin therapy discontinued.

The dogs in Group R were treated conventionally with standard IVCRI of regular insulin, according to a modified previously published protocol (3). The insulin solution was obtained by adding 2.2 U/kg of regular insulin to 48 ml 0.9% NaCl, and was administered in a separate line from the fluids. To overcome the insulin adsorbance to polyvinylchloride surfaces, the insulin solution was allowed to remain in the line for 30 min and was then run through the line (27). After that, the solution was reprepared; the initial dosage was based on the dog's BG concentration at the time in which the IVCRI was started. Subsequent dosage adjustments and the addition of dextrose were carried out based on the BG concentration, measured at 1–2 h intervals (Table 1).

**Table 1:** Sliding scale for the adjustment of intravenous continuous rate infusion of insulin treatment and dextrose supplementation for dogs with diabetic ketoacidosis [modified from Macintire (3)].

**Blood glucose concentration (mg/dL)**	**Fluids**	Rate of administration of insulin solution (mL/h)^*^
>250	0.9% NaCl or Ringer	2
200–250	2.5% dextrose^§^	1.5
150–199	2.5% dextrose^§^	1.5
100–149	5% dextrose^||^	1
<100	5% dextrose^||^	Stop insulin infusion

* *Insulin solution composed of 2.2 U/kg of regular insulin added to 48 mL of 0.9% NaCl*.

§ *2.5% dextrose composed of 25 mL dextrose 50% added to 475 mL of 0.9% NaCl or Ringer*.

|| *5% dextrose composed of 50 mL dextrose 50% added to 450 mL of 0.9% NaCl or Ringer*.

In both groups, the intensive insulin protocol was stopped once the ketosis and acidemia were resolved, hydration status was adequate and appetite returned without vomiting.

During insulin protocols, the serum potassium concentration was corrected as previously described (28). If the serum phosphate concentration decreased to <1.5 mg/dL, hypophosphatemia was corrected by administering potassium phosphate (KPO_4_) as an IVCRI at a rate of 0.01–0.12 mmol phosphate/kg/hour for 6 h and then the phosphatemia was re-evaluated (29, 30). A broad-spectrum antibiotic (Ampicillin/sulbactam, 20 mg/kg every 8 h; Unasyn, Pfizer Italia S.r.l., Latina, IT or Piperacillin/tazobactam, 40 mg/kg every 6 h; Tazocin EF, Pfizer Italia S.r.l., Latina, IT) was administered to all the dogs during hospitalization in order to reduce the risk of complications resulting from pre-existing infections or infections induced by diagnostic and therapeutic procedures, as well as to maximize treatment uniformity. Additional medications, including gastroprotectants, antiemetics, analgesics and/or other antibiotics, were administered as deemed appropriate by the attending clinician according to the clinical condition and concurrent disorders.

### Monitoring Protocol

Blood glucose was measured hourly for the first 24 h using two portable blood glucose meters previously validated for use in dogs (Optium Xceed, Abbott, UK; AlphaTRAK, Zoetis, USA) (31–34), and then every 2–3 h during the intensive insulin protocols. Blood BHB was monitored every 4 h using a portable ketometer, previously validated for dogs (Optium Xceed, Abbott, UK) (35), until the BHB was ≤1.0 mmol/L. The capillary blood samples for the measurement of BG and BHB concentrations were obtained using needles or lancing devices on the inner aspect of the pinna. A venous blood gas analysis, including an electrolyte panel, was carried out, within 5 min of collection, using a blood gas analyzer (ABL 800 Flex, Radiometer Medical ApS, Brønshøj, DK) every 8 h during the first 24 h, and then every 12 h until the acidemia was resolved. Serum phosphate was measured using an automated chemistry analyzer (AU400, Beckman-Coulter/Olympus, O'Callaghan's Mills, Ireland) every 8 h during the first 24 h, and then every 12 h until the DKA was resolved. The venous blood samples were collected by jugular, cephalic or femoral phlebotomy.

Insulin-induced hypoglycemia was defined as a BG concentration <80 mg/dL; hypokalemia and hypophosphatemia were defined as serum potassium ≤3.6 mEq/L and serum phosphate ≤2.65 mg/dL, respectively.

### Definitions of “Resolution Time” of Hyperglycemia, Ketosis, Acidemia, Ketoacidosis, and Length of Hospitalization

The “resolution time” for the variables hyperglycemia, ketosis, and acidemia was calculated starting from “time zero,” which was the time at which insulin protocols were initiated. The time to resolution of severe hyperglycemia was defined as the time interval between “time zero” and the time at which the BG concentration fell to <250 mg/dL. The time to resolution of ketosis was defined as the time interval between “time zero” and the time until the BHB was ≤1.0 mmol/L. The time to resolution of acidemia was defined as the interval between “time zero” and the time at which the venous pH was ≥7.3. The time to resolution of ketoacidosis was defined as the time interval between “time zero” and the time at which ketosis and acidemia had both resolved.

The length of hospitalization (LOH) was defined as the number of hours from admission to discharge from the hospital.

### Statistical Analysis

Continuous variables were considered to be non-parametric because of the small number of cases included in each group. Therefore, the results of the descriptive statistics are reported as median and range (minimum-maximum). The Mann-Whitney *U*-test was used to compare variables between the two insulin groups at the time of admission and at “time zero.” In order to compare the changes in BG concentration and in the BHB from the time of admission and “time zero” within each group, the Wilcoxon signed rank test was used. A *P*-value <0.05 was considered significant. To compare the different variables between dogs with newly diagnosed DM and dogs with known DM, regardless of the type of insulin administered, the Mann-Whitney *U*-test was used. The statistical analysis was carried out using commercially available software (GraphPad Prism 5, GraphPad Software Inc., San Diego, CA).

## Results

A total of 24 cases of DKA in 23 dogs were included in the study; one dog had two DKA events for which it received IM insulin lispro. Eleven cases were managed with IM insulin lispro (Group L) and 13 cases were managed with IVCRI of regular insulin (Group R). Two dogs, one per group, had glycemia ≤ 250 mg/dL, but they were still included in the study due to the presence of acidemia and an increase in BHB concentration (7 mmol/L in dog in Group L, and 6.6 mmol/L in dog in Group R). These two patients had been diagnosed with DM 3 and 5 days before admission and were receiving porcine insulin zinc (0.64 U/kg the dog in Group L and 0.78 U/kg the dog in Group R). It is possible that these two patients have developed DKA due to incorrect insulin dosing or errors in handling insulin. In 20 cases the dogs were discharged from the hospital. Two dogs from Group L died spontaneously, one from sepsis (12 h after admission) and the other from a systemic inflammatory response syndrome secondary to pancreatitis (204 h after admission). Two dogs from Group R were euthanized; the one with severe acute kidney injury was not responding to the treatment protocol and the owner requested euthanasia (50 h after admission); the other was a dog with chronic kidney disease which developed sepsis after the ketoacidosis was resolved and the owner opted for euthanasia (508 h after admission). No dogs were excluded for a low venous pH.

### Signalment, History, Clinical Signs, and Physical Examination Findings

There was no significant difference between the groups with regard to median age, body weight, breed and sexual status (neutered or intact) (Table 2). The median age of all 24 cases was 10 years (range, 5–15 years). The median body weight of all 24 cases was 11.5 kg (range, 3.4–50 kg), and the median body condition score was 6 (range, 3–9). The dog population consisted of eight mixed-breed dogs, four English Setters, two Malteses, two Poodles, and one of each of the following: Alaskan malamute, Cavalier King Charles Spaniel, French Bulldog, Italian Spitz, Labrador Retriever, Pinscher, and Samoyed. Seven dogs were intact males, seven were neutered females, six were intact females, and four were neutered males. The female mixed-breed dog which had two DKA events was intact at the first one and spayed at the second one.

**Table 2:** Baseline data, blood glucose (BG) and beta-hydroxybutyrate (BHB) concentration at admission and at “time zero” in dogs with diabetic ketoacidosis treated with IM insulin lispro (Group L) and treated with IV continuous rate infusion of regular insulin (Group R).

	**Group L**	**Group R**	***P*-value**
Age (years)	10 (6–15)	10 (5–13)	0.6
Body weight (kg)	9 (3.4–46)	16 (3.5–50)	0.22
Male : female	4: 7	7: 6	0.44
Intact males	4/11	3/13	
Neutered males	0/11	4/13	
Intact females	2/11	4/13	
Spayed females	5/11	2/13	
Body condition score	6 (3–8)	6 (3–9)	0.25
BG concentration at admission (mg/dL) [RI 65–115 mg/dL]	478 (105–1,006)	380.5 (191–708)	0.28
BG concentration at “time zero” (mg/dL) [RI 65–115 mg/dL]	431 (227–590)	340.5 (190–456)	0.17
BHB concentration at admission (mmol/L) [RI <2.5 mmol/L]	4.8 (2.6–7.4)	5.3 (3.1–7.1)	0.84
BHB concentration at “time zero” (mmol/L) [RI <2.5 mmol/L]	5.6 (3.6–7)	5.1 (2.8–6.9)	0.62
Serum bicarbonate (mEq/L) [RI 18–23.2 mEq/L]	11 (3.7–17.1)	11.8 (6.9–14.8)	0.82
Venous pH [RI 7.35–7.46]	7.16 (7.08–7.37)	7.14 (7.04–7.29)	0.68
pCO_2_ (mm Hg) [RI 32.7–44.7 mEq/L]	33.7 (13.4–40.9)	25.1 (13.8–32.3)	0.1
Sodium (mEq/L) [RI 143–155 mEq/L]	139 (135–145)	137.5 (130–147)	0.48
Potassium (mEq/L) [RI 3.6–5 mEq/L]	3.8 (2.8–5.5)	4 (2.6–5.2)	0.86
Chloride (mEq/L) [RI 108–118 mEq/L]	98.6 (90.6–112)	102.7 (95.8–114.1)	0.29
Phosphorous (mg/dL) [RI 2.65–5.4 mg/dL]	6.48 (0.84–8.92)	4.2 (1.5–7.3)	0.07
Total Calcium (mg/dL) [RI 9.7–11 mg/dL]	10 (9.1–11.1)	9.1 (8.6–10.6)	**0.01**
Creatinine (mg/dL) [RI 0.75–1.4 mg/dL]	1.09 (0.41–2.68)	0.86 (0.61–7.18)	0.72
Urea nitrogen (mg/dL) [RI 17–48 mg/dL]	95.25 (4.51–239)	48.5 (25.4–147.2)	0.22
Total protein (g/dL) [RI 5.6–7.3 g/dL]	6.9 (4.6–8.3)	6.6 (4.8–7.1)	0.19
Albumin (g/dL) [RI 2.75–3.85 g/dL]	2.88 (2–3.75)	3.26 (1.19–3.56)	0.69
AST (U/L) [RI 15–52 U/L]	57 (16–199)	104.5 (19–177)	0.27
ALT (U/L) [RI 15–65 U/L]	89 (34–767)	110 (29–215)	0.94
ALP (U/L) [RI 12–180 U/L]	1,027 (100–3,024)	400 (123–7,638)	0.86
GGT (U/L) [RI 0–5 U/L]	8 (0.9–150)	9.7 (2.3–70.4)	0.97
Total bilirubin (mg/dL) [RI 0.07–0.33 mg/dL]	0.26 (0.11–1.21)	0.29 (0.17–1.52)	0.54
Cholesterol (mg/dL) [RI 123–345 mg/dL]	459 (259–597)	309.5 (223–509)	**0.03**

*Data are reported as median and range (minimum-maximum). Data were compared with the Mann-Whitney U-tests. Bold indicates significance*.

*RI, reference interval; AST, aspartate transaminase; ALT, alanine transaminase; ALP, alkaline phosphatase; GGT, gamma glutamyltransferase*.

In 14 cases, dogs were newly diagnosed with DM at the time of enrollment into the study (five in Group L and nine in Group R). In 10 cases, the dogs had previously been diagnosed with DM (six in Group L and four in Group R), a median of 1 month (range, 3 days−10 months) prior to enrollment in the study. Nine out of the 10 were receiving insulin therapy, eight dogs with porcine insulin zinc (Caninsulin, MSD, Boxmeer, NL) and one with neutral protamine Hagedorn insulin (Humulin I, Eli Lilly and Co, Sesto Fiorentino, IT). The median insulin dosage at the time of enrollment into the study was 0.55 U/kg (range, 0.1–1.2 U/kg) twice daily.

The clinical signs observed by the owners before admission included polyuria/polydipsia (22/24; 91.6%), anorexia (18/24; 75%), lethargy (17/24; 70.8%), vomiting (13/24; 54.1%), asthenia (10/24; 41.6%), weight loss (9/24; 37.5%), and diarrhea (5/24; 20.8%). Medications administered to the dogs at the time of admission into the hospital included insulin (9/24), trilostane (4/24; Vetoryl, Dechra Veterinary Products Srl, Torino, IT), amoxicillin/clavulanic acid (2/24; Clavaseptin, Vetoquinol Italia S.R.L., Bertinoro, IT), levothyroxine (1/24; Canitroid, Dechra Veterinary Products Srl, Torino, IT), maropitant (1/24; Cerenia, Zoetis Italia SRL, Roma, IT), and ranitidine (1/24; Zantadine, Ceva Salute Animale; Agrate Brianza, IT).

At the time of admission, the most common abnormalities included dull or depressed mentation (17/24; 70.8%), some degree of dehydration (12/24; 50%), abdominal enlargement (12/24; 50%), heart murmurs (7/24; 29.1%), overweight body conditions (BCS ≥7) (6/24; 25%), hyperthermia (>39.0°C) (5/24; 20.8%), muscle atrophy (4/24; 16.6%), congested mucous membranes (3/24; 12.5%), underweight body conditions (BCS ≤3) (2/24; 8.3%), and pale mucous membranes (1/24; 4.1%).

### Clinicopathologic Findings

Upon admission into the hospital and at “time zero,” the median BG concentration, BHB concentration, venous pH and serum bicarbonate concentration were not significantly different between the insulin lispro and regular insulin-treated groups (Table 2).

The median BG concentration in Group L and in Group R upon admission was 478 mg/dL (range, 105–1,006 mg/dL) and 380.5 mg/dL (range, 191–708 mg/dL), respectively (*P* = 0.28). At “time zero,” the median BG concentration was 431 (range, 227–590 mg/dL) and 340.5 mg/dL (range, 190–456 mg/dL) in Group L and in Group R, respectively (*P* = 0.17).

The median BHB concentration in Group L and in Group R upon admission was 4.8 mmol/L (range, 2.6–7.4 mmol/L) and 5.3 mmol/L (range, 3.1–7.1 mmol/L), respectively (*P* = 0.83). At “time zero,” the median BHB concentration was 5.6 mmol/L (range, 3.6–7.0 mmol/L) and 5.1 mmol/L (range, 2.8–6.9 mmol/L) in Group L and in Group R, respectively (*P* = 0.62).

At the time of admission, there were also no significant differences between the two treatment groups with respect to any of the biochemical parameters analyzed, except for serum total calcium (*P* = 0.01) and cholesterol (*P* = 0.03) (Table 2).

The median time interval between the starting the fluid therapy and “time zero” was not significantly different between the insulin lispro group and the regular insulin group: 4 h (range, 3–9 h) and 5 h (range, 1–8 h), respectively (*P* = 0.06).

### Resolution Time of Hyperglycemia, Acidemia, Ketosis, Ketoacidosis, and LOH

Severe hyperglycemia resolved in all 24 cases, and the ketosis and acidemia resolved in 22 cases. The two dogs, one per group, which died prior to resolution of DKA were not included in the analysis.

There were no significant differences in the median time to resolution of hyperglycemia, acidemia, and ketoacidosis between the two groups; however, the median time to resolution of ketosis was significantly shorter in Group L (12 h; range, 4–27 h) as compared to Group R (23 h; range, 10–46 h; *P* = 0.04) (Table 3). In the two dogs that, at the time of admission, had glycemia ≤250 mg/dL, ketosis took 27 h (dog in Group L) and 25 h (dog in Group R) to resolve.

**Table 3:** Time to resolution of hyperglycemia, ketosis, acidemia, and ketoacidosis, and the length of hospitalization in dogs with diabetic ketoacidosis treated with IM insulin lispro (Group L) and treated with IV continuous rate infusion (CRI) of regular insulin (Group R).

	**Group L**	**Group R**	***P*-value**
Resolution time of hyperglycemia (hours)	5 (0–21)	5 (0–9)	0.84
Resolution time of ketosis (hours)	12 (4–27)	23 (10–46)	**0.04**
Resolution time of acidemia (hours)	13 (4–35)	22 (9–80)	0.06
Resolution time of ketoacidosis (hours)	17.5 (4–35)	23.5 (10–80)	0.09
Length of hospitalization (hours)	147 (51–409)	144 (91–342)	0.67

*Data are reported as medians and ranges (minimum-maximum)*.

*Bold indicates significance*.

The median time to resolution of severe hyperglycemia was 5 h in both Group L (range, 0–21 h) and Group R (range, 0–9; *P* = 0.84). The median time to resolution of acidemia in Group L and in Group R was 13 h (range, 4–35 h) and 22 h (range, 9–80 h), respectively (*P* = 0.06). The median time to resolution of ketoacidosis in Group L and Group R was 17.5 h (range, 4–35 h) and 23.5 h (range, 10–80 h), respectively (*P* = 0.09). The median LOH for the 20 cases which were discharged did not differ significantly between Group L (147 h; range, 51–409 h) and Group R (144 h; range, 91–342 h; *P* = 0.67).

There were no significant difference between newly diagnosed and previously diagnosed diabetic dogs with respect to median time to resolution of three variables (hyperglycemia, *P* = 0.68; ketosis, *P* = 0.56; acidemia, *P* = 0.77), median time to resolution of ketoacidosis (*P* = 0.59), and LOH (*P* = 0.19).

The two insulin lispro-treated dogs which spontaneously died had been hospitalized for 12 and 204 h, respectively, at the time of death; the two dogs treated with IVCRI of regular insulin which were euthanized had been hospitalized for 50 and 508 h, respectively, at the time of euthanasia.

Five out of the 11 (45.5%) dogs in Group L failed to have at least a 10% fall in BG concentration by the end of the 1st h. Three dogs required a second loading dose of IM insulin lispro at 0.25 U/kg, one dog required three loading doses every hour, and the last one required five loading doses every hour to achieve an adequate initial response. There was no significant difference when comparing the median BG concentration at “time zero” between the cases in which 1 bolus was able to induce the drop (457 mg/dL; range, 349–590 mg/dL) and cases in which more than one bolus was required (316 mg/dL; range, 227–518 mg/dL) (*P* = 0.39).

### Adverse Insulin Reactions

No local or systemic adverse effects associated with insulin protocols were noted in either group. In group L, intramuscular administration of insulin lispro every 3 h or more frequently did not appear to have caused discomfort and/or pain. Only one dog in Group R developed hypoglycemia during the IVCRI of regular insulin (76 mg/dL), 10 h after “time zero,” but this dog did not show the clinical signs associated with hypoglycemia.

At admission, 6/11 dogs (54.5%) in Group L and 6/13 dogs (46.2%) in Group R had hypokalemia. During insulin protocols, a total of 9/11 dogs (81.8%) in Group L and 10/13 dogs (76.9%) in Group R developed transient hypokalemia (<3.6 mEq/L), despite potassium supplementation; median minimum potassium concentrations did not differ significantly between Group L (3 mEq/L; range, 2.8–3.5 mEq/L) and Group R (2.8 mEq/L; range, 2.1–3.6 mEq/L; *P* = 0.32). Starting from “time zero,” the median time at which the potassium concentration reached the lowest value was significantly different between Group L (8 h; range, 8–24 h) and Group R (16 h; range, 8–56 h; *P* = 0.03). None of the dogs showed clinical signs associated with hypokalemia during insulin protocols.

At admission, the serum phosphate concentrations had been measured in all the dogs in Group L and in 11/13 dogs in Group R. One dog in Group L had severe hypophosphatemia (0.84 mg/dL); this patient received preventive supplementation with potassium phosphate and did not show the clinical signs associated with hypophosphatemia during the protocol with IM insulin lispro. In Group R, 3/11 had mild to moderate hypophosphatemia (1.5, 1.94, and 2.57 mg/dL, respectively), but none of them received preventive supplementation before insulin administration. During the insulin protocols, monitoring of phosphatemia was available in all dogs, except for three dogs in Group L due to technical difficulties. Six out of eight cases (75%) in Group L and 8/13 cases (61.4%) in Group R developed hypophosphatemia. Median minimum phosphorous concentrations did not differ significantly between Group L (1.84 mg/dL; range, 0.29–2.38 mg/dL) and Group R (1.53 mg/dL; range, 0.33–2.47 mg/dL; *P* = 0.66). Starting from “time zero,” the median time at which the serum phosphate concentration had reached the lowest value did not differ significantly between Group L (24 h; range, 8–24 h) and Group R (20 h; range, 8–36 h; *P* = 0.94). None of the dogs showed the clinical signs associated with hypophosphatemia during the insulin protocols.

### Evaluation for the Presence of Concurrent Disorders

Based on the diagnostic protocol, concurrent disorders were identified in 13 dogs (eight in Group L and five in Group R). In Group L, four dogs had pancreatitis, two dogs had Cushing's syndrome, one dog had concurrent Cushing's syndrome and emphysematous cystitis, and one dog had a urinary tract infection. In Group R, one dog had Cushing's syndrome, one dog had a urinary tract infection, one dog had chronic kidney disease, one dog had concurrent pancreatitis and hypothyroidism, and one dog had concurrent pancreatitis and chronic kidney disease.

The diagnosis of pancreatitis was based on abdominal ultrasound (enlarged, irregular, hypoechoic pancreas surrounded by hyperechoic mesentery and mild-to-moderate ascites) and positivity to a canine pancreatic lipase immunoreactivity test.

## Discussion

Intravenous infusion of regular insulin has been the mainstay of treatment of DKA as it causes a more predictable fall in BG and it allows for rapid adjustments (5, 36–38). Nevertheless, IV insulin infusion requires the use of syringe infusion pumps and another venous line to allow the independent handling of fluid replacement and insulin infusion. In human medicine, this complicates the management of patients with DKA due to the limited availability of beds in intensive care units and the higher cost of hospitalization. In veterinary medicine, DKA represents a demanding challenge for clinicians, especially in small outpatient settings in which syringe or infusion pumps are often not available, and frequent biochemical and blood gases monitoring is not possible.

Rapid-acting analogs have been developed to resolve the problems associated with the SC use of regular insulin, such as the delayed peak of activity and the prolonged hypoglycemic effect (39). The use of SC administered rapid-acting analogs has considerably simplified the management of humans with uncomplicated DKA (patients who did not have persistent hypotension after the administration of 1 liter of normal saline, patients not in a comatose state, and those without acute myocardial ischemia, heart failure, end-stage renal disease, hepatic failure, anasarca, dementia, or pregnancy) (18, 19), proving to be similarly effective and safe when compared to IVCRI of regular insulin (18–21).

In veterinary medicine, the use of rapid-acting analogs has been evaluated in dogs and cats with DKA, but only using protocols based on IVCRI (22–25). To the authors' knowledge, the use of rapid-acting analogs *via* other route of administration for the treatment of dogs with DKA has not yet been investigated.

The aim of this study was to evaluate the efficacy and safety of the IM administration of insulin lispro for the treatment of DKA in dogs. The median time to resolution of hyperglycemia, and the LOH were similar in Group L and in Group R (*P* = 0.66 and *P* = 0.67, respectively). However, the median time to resolution of ketosis was significantly shorter in Group L (12 h; range, 4–27 h) as compared to Group R (23 h; range, 10–46 h; *P* = 0.04). The median times to resolution of acidemia and ketoacidosis were both shorter in the group of dogs treated with IM insulin lispro, although these differences were not significant (*P* = 0.06 and *P* = 0.09, respectively). We hypothesize that, in studying a larger group of cases, these times may be significantly shorter in dogs treated with IM insulin lispro.

The median times to resolution of ketosis (12 h), acidemia (13 h) and ketoacidosis (17.5 h) in dogs treated with IM insulin lispro were shorter than the times previously reported in dogs treated with IVCRI of insulin lispro (26, 26, and 52 h, respectively) and in dogs treated with IVCRI of insulin aspart (22, 21, and 28 h, respectively) (22, 23). However, direct comparison with similar studies, due to different study designs, should be evaluated with caution. The results of the present study demonstrated the efficacy of the IM administration of insulin lispro in the treatment of canine DKA and suggested the hypothesis that this route of administration could lead to results similar to those of IV infusion.

In Group L, a single IM bolus at 0.25 U/kg of insulin lispro was effective in inducing a drop of at least 10% in BG concentration by the end of the 1st h in only six out of eleven dogs (54.5%). This finding suggested the possibility of administering a higher initial bolus (e.g., 0.3 U/kg instead of 0.25 U/kg) in order to obtain a glycemic drop in a greater percentage of patients. However, the safety of this approach remains to be studied.

With regard to the safety of insulin administration in the population in the present study, side effects occurred with a similar frequency in dogs treated with IM insulin lispro and in dogs treated with IVCRI of regular insulin. A single asymptomatic hypoglycemic event occurred in one dog in Group R during the insulin protocol. In the study by Sears et al. (22), two out of six dogs developed hypoglycemia during IVCRI of insulin lispro; in the study of Walsh et al. (23), the IVCRI administration of insulin aspart was discontinued because BG was <100 mg/dL in four out of six dogs (in two of them discontinuation occurred four and six times, respectively).

At admission, hypokalemia was present in 54.5% and in 46.2% of the dogs in Group L and Group R, respectively. During insulin protocols, transient asymptomatic hypokalemia occurred in 81.8% and in 76.9% of dogs in Group L and in Group R, respectively. The median minimum potassium concentrations did not differ significantly between the groups (*P* = 0.32); however, it occurred significantly earlier in patients in Group L (8 h) than in patients in Group R (16 h; *P* = 0.03). Although hypokalemia which develops during DKA rarely becomes symptomatic, the results of the present study supports the use of potassium supplementation from the 1st h of insulin therapy, but only if urinary production is adequate and frequent assessments of the kalemia are possible.

During insulin protocols, asymptomatic hypophosphatemia occurred in 75% and in 61.4% of dogs in Group L and in Group R, respectively. The median minimum serum phosphate concentrations did not differ significantly between the groups (*P* = 0.66). None of the dogs, with the exception of the dog in Group L which had severe hypophosphatemia (0.84 mg/dL) at the time of admission, required phosphate supplementation. In dogs with DKA, regardless of the type of insulin and the route of administration, it is therefore advisable to frequently monitor the phosphate concentration; another option could be phosphate supplementation in all dogs treated for DKA, except for patients with hyperphosphatemia or with kidney disease.

This study, which aimed to ascertain the safety and effectiveness of a new intramuscularly insulin lispro protocol, has some limitations. The first limitation was that the number of cases enrolled was not based on a power calculation and it was relatively small. This is a common limitation in studies relating to the topic and it cannot be excluded that future studies carried out on a larger number of cases could confirm or refute the results of the present study. Other limitations were the lack of randomization and the temporal distance between the two insulin protocols; in fact, cases in the control group (Group R) were enrolled between April 2015 and May 2017 while cases in the test group (Group L) were enrolled between June 2018 and August 2019. The different experiences of the clinicians in managing the DKA could also have influenced the success of treatment and the outcome. However, the extreme closeness between the two periods makes this bias unlikely. Another limitation was the heterogeneity of the population with regard to the presence of concurrent disorders; these determined a different severity and duration of insulin resistance and could have interfered with the response to the treatment protocol. It would be interesting to stratify illness severity by mortality risk (APPLE score) (40) to describe and compare the two populations, but this was not possible due to the lack of data, especially in the control population. However, the homogeneity between the groups at the time of admission, as regards the variables under study (BG concentration, pH value and the degree of ketosis and acidemia), should increase the reliability of the comparison. The authors hypothesize that the differences in serum total calcium and cholesterol concentrations between the two treatment groups at the time of admission were either a result of chance or dependent on concomitant disorders. Moreover, the population characteristics in this study were very similar to those of other studies of canine DKA (22, 23, 26, 41), suggesting that this small population was representative of dogs with spontaneous DKA.

Another limitation is that the “time zero” was not standardized, but it was defined according to the clinician's subjective assessment of the degree of dehydration. Similarly, the type of crystalloids administered for the fluid resuscitation was also determined by the attending clinician according to his clinical judgment. Another limitation is the inaccuracy with which the resolution times of ketosis and acidemia were estimated; in fact, blood BHB was monitored every 4 h and a venous blood gas analysis was performed every 8–12 h. The loss of information between one evaluation and the next may have overestimated the result. Furthermore, the use of two different portable blood glucose meters, which has been dictated by the availability of the strips, is a confounding factor and may have vitiated the results.

In conclusion, results of the present study demonstrated that the new protocol based on the IM administration of insulin lispro was effective and safe for treatment of DKA in dogs. Larger future studies should build on these findings to additionally validate or modify the treatment protocol reported here. Finally, in order to establish which is the most effective and safe route of administration of insulin lispro to manage canine DKA, studies that make a direct comparison between the two treatment protocols would be appropriate.

## Data Availability Statement

The raw data supporting the conclusions of this article will be made available by the authors, without undue reservation.

## Ethics Statement

The animal study was reviewed and approved by the Animal Welfare Committee (COBA) of the Alma Mater Studiorum—University of Bologna (Bologna DL 26/2014, Project 792). Written informed consent was obtained from the owners for the participation of their animals in this study.

## Author's Note

The content of this manuscript has been published as part of the thesis of EM Chetoacidosi diabetica nel cane e nel gatto: nuove prospettive terapeutiche e strumenti di monitoraggio (2019) [dissertation/PhD thesis]. [Bologna (Italy)]: University of Bologna.

## Author Contributions

EM designed the study, analyzed data, co-wrote, and edited the manuscript. FA and GG applied the protocol in dogs with DKA and co-wrote the manuscript. MG edited the manuscript. FF designed the study and edited the manuscript. All authors contributed to read and approved the final manuscript.

## Conflict of Interest

The authors declare that the research was conducted in the absence of any commercial or financial relationships that could be construed as a potential conflict of interest.

## Abbreviations

BG: blood glucose
BHB: beta-hydroxybutyrate
DKA: diabetic ketoacidosis
DM: diabetes mellitus
IVCRI: intravenous continuous rate infusion
LOH: length of hospitalization
NaCl: sodium chloride.
